# LKB1 regulates ILC3 postnatal development and effector function through metabolic programming

**DOI:** 10.3389/fimmu.2025.1587256

**Published:** 2025-06-05

**Authors:** Huasheng Zhang, Linfeng Zhao, Qingbing Zhang, Lin Hu, Xiaohui Su, Jiping Sun, Lei Shen

**Affiliations:** ^1^ Center for Immune-Related Diseases at Shanghai Institute of Immunology, Ruijin Hospital, Shanghai Jiao Tong University School of Medicine, Shanghai, China; ^2^ Department of Immunology and Microbiology, Key Laboratory of Cell Differentiation and Apoptosis of Chinese Ministry of Education, Shanghai Jiao Tong University School of Medicine, Shanghai, China; ^3^ Shanghai Key Laboratory of Tumor Microenvironment and Inflammation, Shanghai Jiao Tong University School of Medicine, Shanghai, China

**Keywords:** group 3 innate lymphoid cells (ILC3s), Liver Kinase B1 (LKB1), metabolic programming, intestinal immune homeostasis, inflammation

## Abstract

**Introduction:**

Group 3 Innate Lymphoid Cells (ILC3s) are important for maintaining intestinal homeostasis and host defense. Emerging studies have shown that metabolic regulation plays a crucial role in regulating ILC3 activation and function. However, the role of Liver Kinase B1 (LKB1), a key metabolic regulator, in regulating ILC3 function and intestinal immunity remains poorly understood.

**Methods:**

To investigate the role of LKB1 in intestinal ILC3s, we generated LKB1 conditional knockout mice by crossing *Rorc* ^cre^ and *Stk11* ^flox/flox^ mice. Cell number and cytokine production was examined using flow cytometry. *Citrobacter rodentium* infection model were used to determine the role of LKB1 in intestinal defense. RT-qPCR, flow cytometry and immunohistochemistry were used to assess the intestinal inflammatory responses.

**Results:**

In this study, we show that LKB1 is essential for ILC3 postnatal development, effector function, and intestinal immunity. LKB1-deficient mice exhibit a marked decrease in ILC3 number at 2 -3 weeks after birth. Ablation of LKB1 in ILC3s results in diminished IL-22 production and less protection against *Citrobacter rodentium* infection. Moreover, LKB1 deficiency leads to impaired cell metabolism, as indicated by reduced glycolysis and oxidative phosphorylation and less mitochondrial mass. Together, our data demonstrate that LKB1 promotes ILC3 postnatal development and effector function to maintain intestinal immune homeostasis.

**Discussion:**

Our findings reveal that LKB1 is a key regulator of intestinal ILC3 development, function, and metabolism, thereby linking metabolic control to intestinal immune homeostasis and offering potential therapeutic implications.

## 1 Introduction

Innate lymphoid cells (ILCs) are tissue-resident innate immune cells with lymphoid morphology but lack rearranged antigen receptors (1, 2). Group 3 ILCs (ILC3s) are characterized by the expression of transcription factor retinoic acid-related orphan receptor gamma t (RORγt) and production of cytokines interleukin (IL) -22 and IL-17 (3). ILC3s are highly enriched in the intestinal mucosa, where they maintain the tissue barrier integrity and contribute to the host defense against pathogens (4). Disrupted ILC3 responses are associated with the susceptibility to inflammatory bowel disease (IBD) and gut bacterial infection. Studies indicate that IL-22 mediates immune defense and limits pathogen expansion by promoting antimicrobial peptides production (5), while IFN-γ, TNF-α and IL-17 are thought to promote intestinal inflammation and contribute to the pathogenesis of colitis (5–7).

Emerging studies demonstrate that metabolic programming controls ILC3 activation and effector function (8–10). For instance, glycolysis promotes effector cytokine IL-22 production in activated ILC3s (9), whereas lipid oxidative phosphorylation suppresses IL-22 secretion (10). Metabolic regulator mTOR complex 1 (mTORC1) has been shown to drive glycolysis in activated ILC3s, therefore to support cell proliferation and cytokine production (11, 12). Cellular metabolism is also important for ILC3 fate determination. ILC3 can acquire a long-term activated phenotype following *C. rodentium* infection, called “trained ILC3s” (13). The metabolic shift from glycolysis and glutamine metabolism to increased TCA cycle and OXPHOS is thought to be responsible for this process (13). Given the complexity of metabolic regulation, the metabolic pathways governing ILC3 activation and function remain largely unknown.

Liver Kinase B1 (LKB1), a serine/threonine kinase, is a critical regulator of cell energy homeostasis and cell metabolism (14–16). Recent studies have shown that LKB1 plays an important role in regulating multiple immune cell activation mechanisms and function through metabolic reprograming in T cells, Dendritic cells, Hematopoietic stem cells, and Group 2 Innate lymphoid cells (17–22). However, the role of LKB1 in regulating ILC3 function remains undefined. In this study, we found that LKB1 regulated intestinal ILC3 postnatal development, effector function, and cell metabolism. Loss of LKB1 resulted in a significant reduction in intestinal ILC3s, which was attributed to increased apoptosis and decreased proliferation. Deletion of LKB1 in ILC3s also led to diminished IL-22 production, leading to impaired gut defense against *C. rodentium* infection. Moreover, LKB1-deficient ILC3s exhibited significantly reduced expression of genes involved in energy metabolism, particularly those associated with glycolysis and oxidative phosphorylation. Collectively, our data demonstrate that LKB1 is required to sustain intestinal ILC3 function and gut immune defense.

## 2 Materials and methods

### 2.1 Mice

All mice used in this study were on the C57BL/6J background. Wild-type mice (C57BL/6) were purchased from Shanghai Laboratory Animal Center (SLAC). *Rag1^-/-^* mice (Strain: 002216), *Stk11tm1.1Sjm* (*Stk11* ^f/f^) mice (Strain: 014143), *Id2 tm1.1(cre/ERT2)Blh* (*Id2* ^cre-ERT2^) mice (Strain: 016222) and *Rorc* ^cre^ mice (Strain: 022791) were purchased from the Jackson laboratory (Bar Harbor, Me). All mice were bred and maintained at accredited animal facilities under specific-pathogen-free conditions in individually ventilated cages on a strict 12-hour day–night cycle with a regular chow diet. Mice used in this study were 6–8 weeks old and sex-matched, unless otherwise indicated in the text. Six-week-old *Id2* ^cre-ERT2^ *Stk11* ^f/f^ mice were injected intraperitoneally with tamoxifen (2 mg/day per mouse) for 5 days to knock down *Stk11* expression in the ILC lineage. All animal experiments were performed in compliance with the Guide for the Care and Use of Laboratory Animals, approved by Shanghai Jiao Tong University School of Medicine Institutional Animal Care and Use Committees (IACUC) and performed according to guidelines for the use and care of laboratory animals as provided by Shanghai Jiao Tong University School of Medicine Institutional Animal Care and Use Committees (IACUC).

### 2.2 *Citrobacter rodentium* infection

*C. rodentium* strain, DBS100 (ATCC) was grown in 2 mL of LB broth at 37°C for 8–10 h. Then 40 μL of culture was added to 200 mL of LB broth to grow for another 8–10 h at 37°C. Bacteria were pelleted at 5,000g for 8 min and then resuspended in 8 ml of sterile tri-distilled water. Mice were inoculated with 1 × 10^10^ CFU by gastric gavage. Feces and tissue samples were collected and weighed. Mice will be sacrificed when the body weight loss is over 25% of initial weight.

### 2.3 *Citrobacter rodentium* titers in mouse feces, colon and spleen

Mice were euthanized, and fecal pellets, colon, and spleen tissues were collected. Feces were resuspended in sterile PBS (1 mL), while colon and spleen tissues were homogenized in 1 mL sterile PBS. Serial dilutions of fecal, colon, and spleen samples were plated on MRS agar and incubated anaerobically at 37°C for 48 hours. Colony-forming units (CFUs) of *Lactobacillus acidophilus* were counted. The bacterial titer was calculated as CFUs per gram of feces, colon, or spleen, normalized by the sample weight. Results are expressed as log-transformed CFUs/g.

### 2.4 Isolation of intestinal lamina propria lymphocytes

For isolation of intestines lamina propria immune cells, fat tissues were removed carefully from the intestines and then tissues were cut into pieces and washed in 10 mL of PBS containing 1 mM dithiothreitol (Sigma) and 30 mM EDTA for 10 minutes, and then in 10 mL of PBS containing 30 mM EDTA for another 10 minutes with shaking at 100 xg. Then tissues were digested in RPMI 1640 medium containing DNase I (150 μg/mL) and collagenase VIII (400 U/mL) at 37°C for 1.5h (for large intestine) or 1h (for small intestine). After vigorous shaking, digested tissues were filtrated through a 70 μm cell strainer, intestine mononuclear cells were harvested from the interphase of a 40% and 80% Percoll gradient after a spin at 1200 xg for 20 min at room temperature.

### 2.5 Flow cytometry and cell sorting

Cells were stained with fixable viability dye (BD Biosciences) for 10 min at room temperature to discriminate dead cells and incubated with anti-CD16/32 (Biolegend) for 15 min at 4°C to block FcR. For surface staining, cells were stained with antibody cocktails diluted in 100 μL of FACs buffer for 30 min at 4°C in the dark. For intracellular cytokines detection, cells were stimulated with PMA (50 ng/mL, Sigma-Aldrich) and ionomycin (500ng/mL, Sigma-Aldrich) for 4h, and brefeldin A (2 μg/mL, Sigma-Aldrich) was also added. After surface staining, cells were fixed and permeabilized by IC fixation buffer (Invitrogen) for 45 min at 4°C and stained with intracellular antibody cocktails in 100 μL permeabilization buffer (Invitrogen) for 45 min at 4°C in the dark. For transcription factor staining, cells were fixed and permeabilized by Foxp3 Fixation/Permeabilization buffer (Invitrogen) for 45 min at 4°C and stained with antibody diluted in 100 μL of permeabilization buffer (Invitrogen) for 45 min at 4°C in the dark. For phosphorylation staining, cells were fixed in Phoflow™ fixation buffer I (BD Biosciences) for 30 min at 37°C, and then stained with phosphorylated antibodies for 30 min at room temperature prior to addition of the secondary antibody for 30 min at room temperature. For proliferation assay analyses, cells were fixed and permeabilized by Foxp3 Fixation/Permeabilization buffer (Invitrogen) for 45 min at 4°C and stained with anti-Ki67 diluted in 100 mL of permeabilization buffer (Invitrogen) for 45 min at 4°C in the dark. For apoptosis assay analyses, cells were stained with anti- active Caspase 3 antibodies (BD Horizon™ Clone: C92-605) according to the manufacturer’s instructions. Stained cells were analyzed on a BD FACSymphony™ A5 or LSRFortessa™, and the data were analyzed by FlowJo version 10 software. For cell sorting, ILC3s from large intestine and small intestine were sorted as Lin^-^CD45.2^mid^CD90.2^hi^KLRG1^-^ cells using a BD FACS AriaIII, Lin = CD3e, Gr1, CD11b, CD11c, CD5, CD19, and NK1.1.

### 2.6 Strategy to clear gut microbiota in neonatal mice

Pregnant breeding mice (gestational age >2 weeks) were administered antibiotic cocktails (ABX) (vancomycin, 0.5 g/L; neomycin sulfate, 1 g/L; metronidazole, 1 g/L; ampicillin, 1 g/L) in their drinking water. This treatment regimen was maintained until the offspring mice reached 3 weeks of age (23).

### 2.7 Histologic analysis

Middle colon tissues were dissected and fixed in 4% paraformaldehyde and embedded in paraffin. Sectioned were stained with hematoxylin and eosin and examined by light microscopy. Images were acquired using the OLYMPUS BX53 system.

### 2.8 Cellular metabolism analysis

For analysis of mitochondrial mass or mtROS production, cells were incubated in RPMI 1640 containing MitoTracker Deep Red (100 nM, Thermo Fisher Scientific) for 20 min or MitoSOX™ Red (3.3 μM, Thermo Fisher Scientific, M36008) for 10 min at 37 °C, respectively. Cells were then stained with surface antibodies and analyzed by flow cytometry using FACSymphony™A5. Data were analyzed using FlowJo version 10 software.

### 2.9 Seahorse cellular metabolic assays

Intestine ILC3s were sorted from *Stk11* ^f/f^ and *RORc* ^cre^ *Stk11* ^f/f^ mice and cultured *in vitro* for 2 days. Cells were plated at 90,000 cells per well, and OCR and ECAR were measured in Seahorse XF media (RPMI 1640 containing 5 mM glucose, 2 mM L-glutamine and 1mM sodium pyruvate) under basal conditions, in response to 1 μM oligomycin, 1.5 μM fluror-carbonyl cyanide phenylhydrazone (FCCP) and 100 nM rotenone 1 μM antimycin A using Mito stress test kit (Agilent). All oxygen consumption and extracellular acidification measurements were conducted using an Agilent Seahorse XF96 Analyzer.

### 2.10 RNA sequencing and data analysis

Intestine ILC3 were sorted from *Stk11* ^f/f^ and *RORc* ^cre^ *Stk11* ^f/f^ mice. Total RNA was extracted by TRIzol reagent (Invitrogen). RNA was qualified and quantified using a Nano Drop and Agilent 2100 Bioanalyzer. Limited RNA (more than 200 pg) was amplified and then reverse transcribed to cDNA for downstream library construction. The SMART-Seq HT Kit (Takara Bio USA) was used to generate high-quality, full-length cDNA. Sequencing data were aligned to the mouse reference genome (version mm10). Quantitated relative mRNA expression levels (FPKM) were calculated on the basis of exon regions using Cufflinks and the mm10 reference genome annotations. Protein-coding genes were shown by volcano plots and used for Gene Set Enrichment Analysis (Broad Institute). Differentially expressed genes identified by DESeq2 (fold change >1.5, p < 0.05) were highlighted in the volcano plot and used for Gene Ontology (GO) analysis. Heat maps of the normalized gene counts were generated using Morpheus. RNA-sequencing data have been deposited in GEO under the primary accession code GSE284792.

### 2.11 RNA extraction and quantitative real-time

Total RNA was isolated from mouse intestines or sorted intestine ILC3s using TRIzol reagent (Invitrogen). cDNA was synthesized using PrimeScript™ RT master Kit (Takara). qRT-PCR was performed using SYBR™ Select Master Mix (Themo Fisher Scientific), and reactions were run with QuantStudio Real-Time PCR software V1.3 (Thermo Fisher Scientific) on a real-time PCR system (AB applied biosystems ViiA7). All primers were purchased from Sangon Biotech.

### 2.12 Statistical analysis

Data were analyzed by two-tailed unpaired or paired Student’s t test for comparing two groups, and by two-way analysis of variance analysis (ANOVA) followed by Tukey’s multiple comparisons test for comparing multiple groups. All data were analyzed using GraphPad Prism 8.0 program. Data points represent biological replicates and are shown as the mean ± SEM. p values are noted in each figure and p values of less than 0.05 are considered statistically significant.

## 3 Results

### 3.1 LKB1 is associated with intestinal ILC3 activation

**Figure 1:**
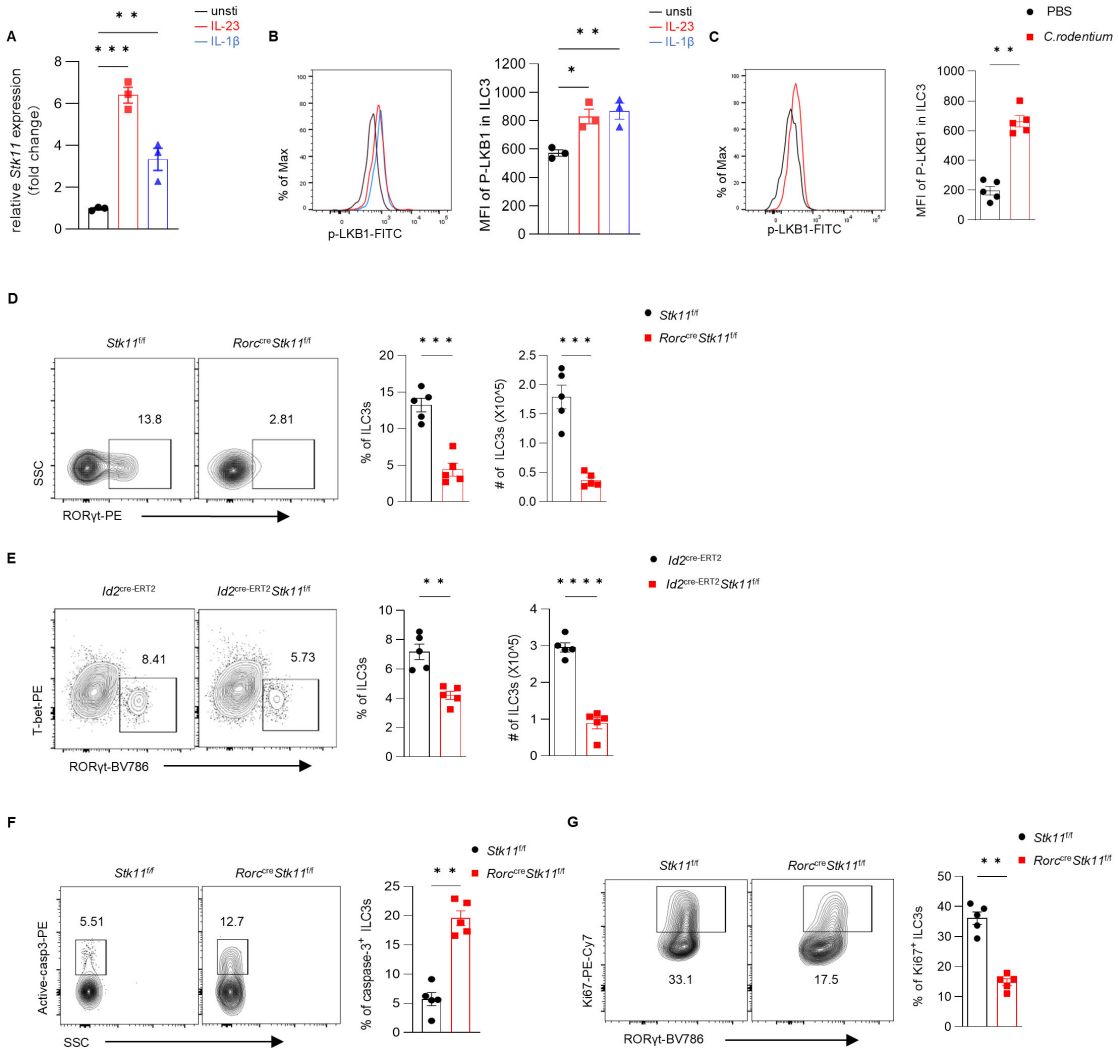
LKB1 is required for intestinal ILC3 homeostasis. **(A, B)** ILC3 sorted from the large intestine (LI) lamina propria lymphocytes (LPLs) of 8-week-old C57BL/6 mice were stimulated with rmIL-23 (25 ng/ml) or rmIL-1β (25 ng/ml) for 24 h **(A)** or 1h **(B)**. **(A)** qRT-PCR analysis of *Stk11* mRNA expression (n = 3). **(B)** Phosphorylated LKB1 (P-LKB1) expression by flow cytometry (n = 3). **(C)** 8-week-old C57BL/6 mice were orally inoculated with *C*. *rodentium* (1*10^10^ CFU). P-LKB1 expression in LI ILC3s were analyzed by flow cytometry on day 6 (n = 5). **(D, E)** Frequency and absolute numbers of ILC3s in LI from *Stk11* ^f/f^ and *Stk11^ΔRorc^* mice (n = 5) **(D)**, or from *Id2* ^cre−ERT2^ and *Id2* ^cre−ERT2^ *Stk11* ^f/f^ mice (n = 5) **(E)**. Cells were gated on live CD45^+^ Lin^-^ CD127^+^ cells. Lin = CD3e, Gr1, CD11b, CD11c, CD5, CD19, and NK1.1. **(F)** Frequency of active Caspase 3 ILC3s in LI (n = 5). **(G)** Frequency of Ki-67^+^ ILC3s in LI (n = 5). Data are representative of three **(A-G)** independent experiments shown as mean ± SEM. Statistical significance was tested by two-tailed unpaired Student’s *t*-test in **(A-G)**. *P < 0.05,**P < 0.01, ***P < 0.001.

To determine whether LKB1 plays a role in intestinal ILC3 function, we first examined the expression level of LKB1 in activated ILC3s. LKB1 expression was significantly increased in ILC3s upon stimulation with IL-1β or IL-23 *in vitro* (**Figure 1A**; **Supplementary Figure S1A**). In addition, activated ILC3s exhibited enhanced LKB1 phosphorylation (Ser428) (**Figure 1B**). To further explore the role of LKB1 in intestinal inflammation, we utilized *Citrobacter rodentium (C. rodentium)* infection—a widely accepted and physiologically relevant model for studying ILC3-mediated mucosal immune responses. Notably, we observed a marked increase in LKB1 phosphorylation in ILC3 isolated from *C. rodentium*-infected mice (**Figure 1C**; **Supplementary Figure S1B**). These findings suggest that LKB1 activation is associated with ILC3 activation and prompt us to hypothesize that LKB1 might regulate ILC3 activation.

### 3.2 LKB1 deficiency leads to reduced numbers of ILC3s

To investigate the impact of LKB1 on intestinal ILC3 homeostasis, we generated *RORc* ^cre^ *Stk11* ^f/f^ (*Stk11^ΔRorc^*) mice to delete LKB1 in ILC3s. The number of ILC3 in the intestinal lamina propria was significantly decreased in *Stk11^ΔRorc^* mice compared to control *Stk11* ^f/f^ mice in large intestine and small intestine (**Figure 1D**; **Supplementary Figure S2A**). Given that RORγt expression starts in ILC3 progenitors in the fetal liver, the reduction of ILC3 number in adult *Stk11^ΔRorc^* mice might be caused by the impairment of ILC3 development or the survival of mature intestinal ILC3s. To determine whether LKB1 plays a role in mature intestinal ILC3s, we used *Id2* ^cre-ERT2^ *Stk11* ^f/f^ mice, in which LKB1 depletion can be induced by tamoxifen administration in adult mice. Consistently, KO mice had fewer ILC3 cells in both large intestine and small intestine compared with WT mice (**Figure 1E**; **Supplementary Figure S2B**). Because mature ILC3 cell number is maintained predominantly by cell proliferation and survival, we next examined ILC3 proliferation and survival in these mice. Interestingly, *Stk11^ΔRorc^* mice showed increased cell apoptosis, as indicated by higher active caspase-3 levels, and reduced proliferation, as shown by lower intracellular Ki67 staining (**Figures 1F, G**). These findings suggest that LKB1 is required for ILC3 maintenance.

### 3.3 LKB1 promotes ILC3 postnatal development

**Figure 2:**
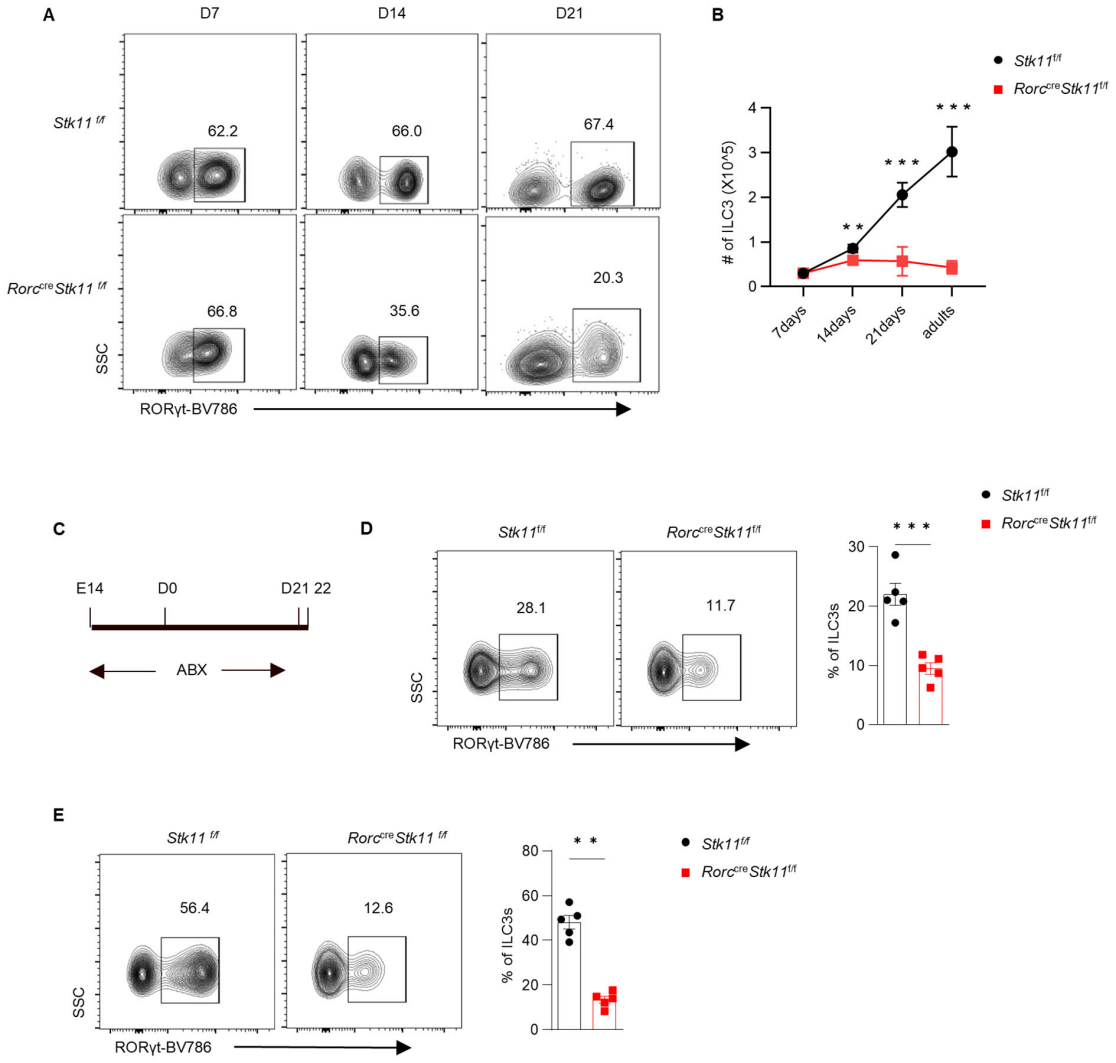
LKB1 controls ILC3 postnatal development. **(A)** Representative flow cytometric plots showing frequency of ILC3s in large intestine (LI) from *Stk11* ^f/f^ and *Stk11^ΔRorc^* mice at the indicated time points after birth. **(B)** Quantification of absolute numbers of ILC3s in LI. **(C)** Strategy to clear gut microbiota in neonatal *Stk11^ΔRorc^* and *Stk11* ^f/f^ mice. The breeding mice that had been pregnant for more than two weeks were administered antibiotic cocktails (ABX) in their drinking water until the offspring mice reached three weeks old. **(D, E)** Frequency of ILC3s in large intestine (LI) **(D)** and small intestine (SI) **(E)** from *Stk11* ^f/f^ and *Stk11^ΔRorc^* mice which were treated with ABX on 3 weeks after birth (n = 5). Data are representative of three **(A-E)** independent experiments shown as mean ± SEM. Statistical significance was tested by two way ANOVA followed by Tukey’s multiple comparisons test in **(B)** or two-tailed unpaired Student’s *t*-test in **(D, E)**. **P < 0.01, ***P < 0.001.

To further explore what stage LKB1 plays a role during ILC3 development, we investigated the ontogeny of ILC3s in *Stk11^ΔRorc^* mice. Consistent with previous reports, ILC3s from control *Stk11* ^f/f^ mice expanded rapidly within intestine tissues during the first three weeks after birth, which was presumably due to the colonization of microbiota and exposure to other environmental stimuli (23). Notably, 1-week-old *Stk11^ΔRorc^* mice had similar cell number of RORγt^+^ ILC3s to the control mice. However, 2-week-old *Stk11^ΔRorc^* mice started to show decreased frequency of ILC3s in small intestine, whereas the decrease of ILC3s became more striking at 3 weeks after birth (**Figures 2A, B**). Given that commensal microbiota start colonization in the gut after birth, we hypothesized that gut microbiota might contribute the reduction of ILC3s in *Stk11^ΔRorc^* mice. To test this hypothesis, antibiotics were used to clear gut microbiota in pregnant mice and continued for three weeks post-partum (**Figure 2C**). Surprisingly, depletion of gut microbiota by antibiotics did not restore the ILC3 number in *Stk11^ΔRorc^* mice (**Figures 2D, E**), suggesting a microbiota-independent downregulation of ILC3s in the absence of LKB1. Together, these data indicate that LKB1 plays an essential role in sustaining postnatal ILC3 development.

### 3.4 LKB1 regulates ILC3 effector function and gut immunity to *C. rodentium*

**Figure 3:**
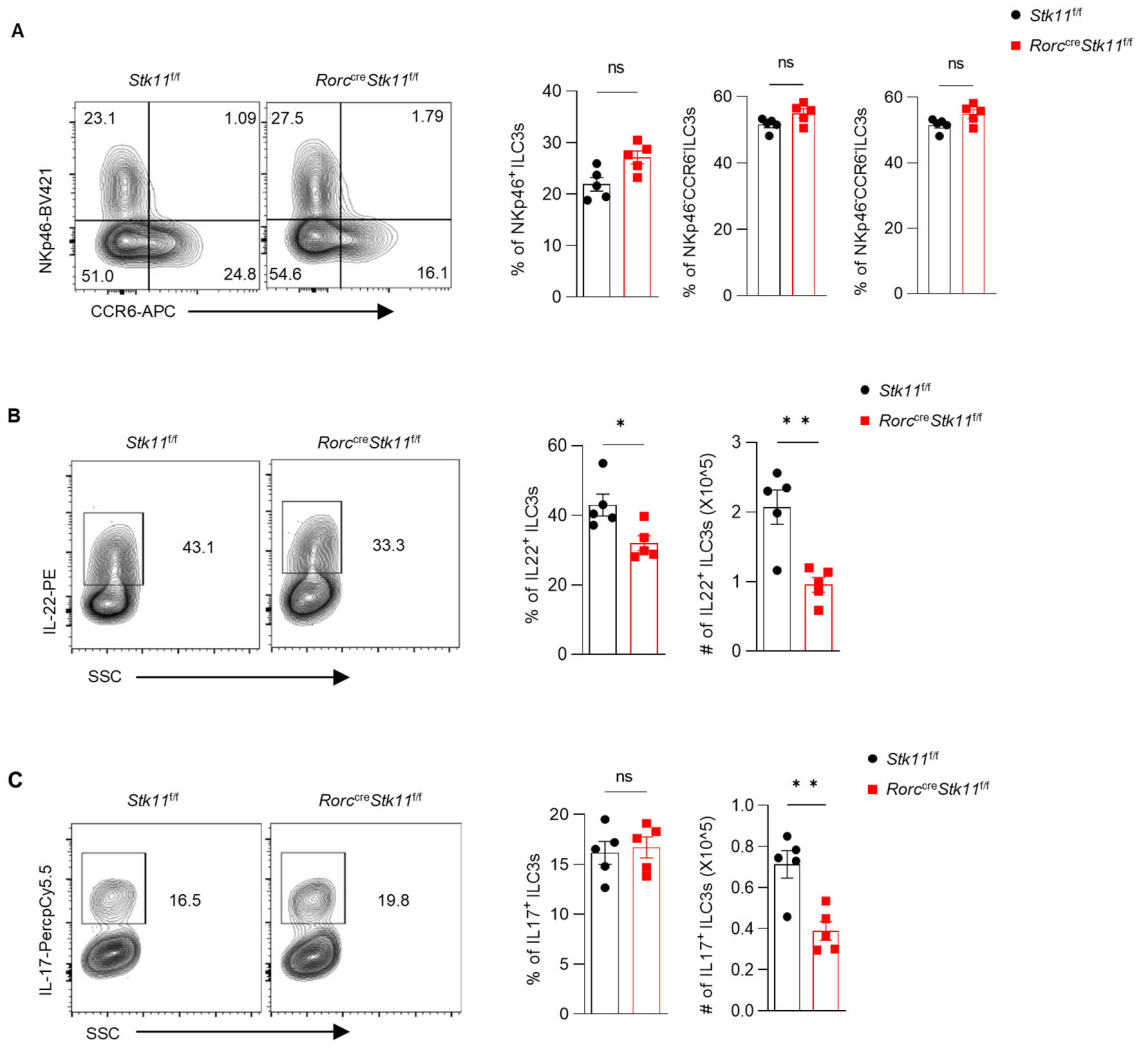
Ablation of LKB1 in ILC3s results in diminished cytokine production. **(A)** Frequency and absolute numbers of NKp46^+^ and CCR6^+^ ILC3s in large intestine (LI) (n = 5). **(B, C)** Frequency and absolute numbers of IL-22^+^ILC3s **(B)** and IL-17^+^ILC3s **(C)** in LI (n = 5). Cells were gated on live CD45^+^ Lin^-^ CD127^+^ Rorγt^+^ lymphocytes **(A-C)**. Data are representative of three **(A-C)** independent experiments shown as mean ± SEM. Statistical significance was tested by two-tailed unpaired Student’s *t*-test in **(A-C)**. *P < 0.05, **P < 0.01.

**Figure 4:**
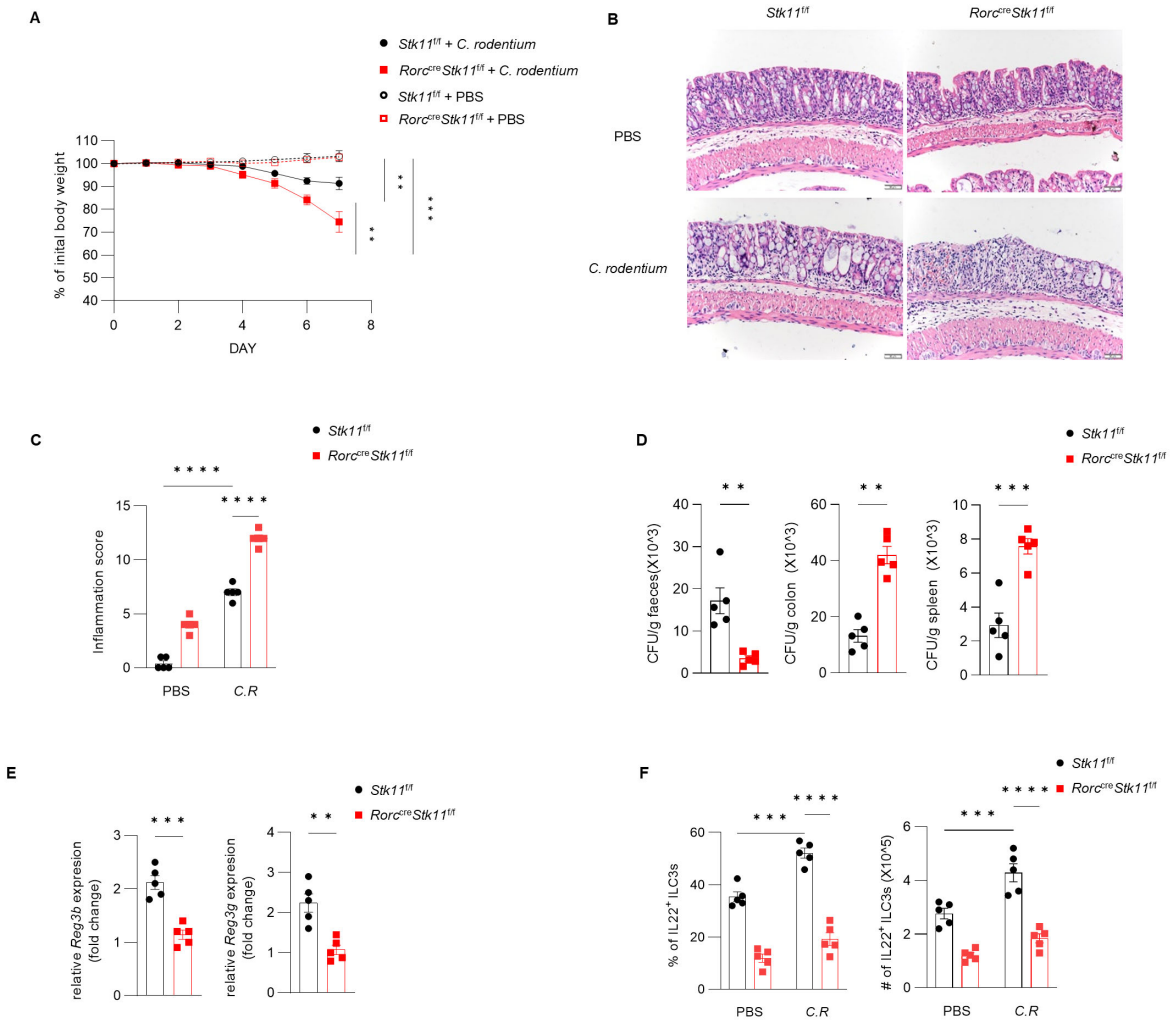
Ablation of LKB1 in ILC3s compromises host defense against *C*. *rodentium*. **(A–D)** 8-week-old *Stk11^ΔRorc^* mice and their wild-type *Stk11* ^f/f^ littermates were orally inoculated with *C*. *rodentium* (2*10^9^ CFU) and analyzed on day 7 after infection. **(A)** Body-weight changes (n = 5). **(B)** Histological analysis of representative colons from *Stk11* ^f/f^ and *Stk11^ΔRorc^* mice. Scale bars represent 50 mm. **(C)** Histological inflammation scoring of colonic tissues from *Stk11* ^f/f^ and *Stk11^ΔRorc^* mice. **(D)** *C*. *rodentium* titers in feces, colon, and spleen from mice (n = 5). **(E)** Frequency and absolute numbers of IL-22^+^ILC3s in large intestine (LI) from *Stk11* ^f/f^ and *Stk11^ΔRorc^* mice (n = 5). **(F)** The mRNA expression of antimicrobial proteins *Reg3g* and *Reg3b* in the colon of mice (n = 5). Data are representative of three **(A-E)** independent experiments shown as mean ± SEM. Statistical significance was tested by two way ANOVA followed by Tukey’s multiple comparisons test in **(A)**, or two-tailed unpaired Student’s *t*-test in **(C-E)**. **P < 0.01, ***P < 0.001, ****P < 0.0001.

**Figure 5:**
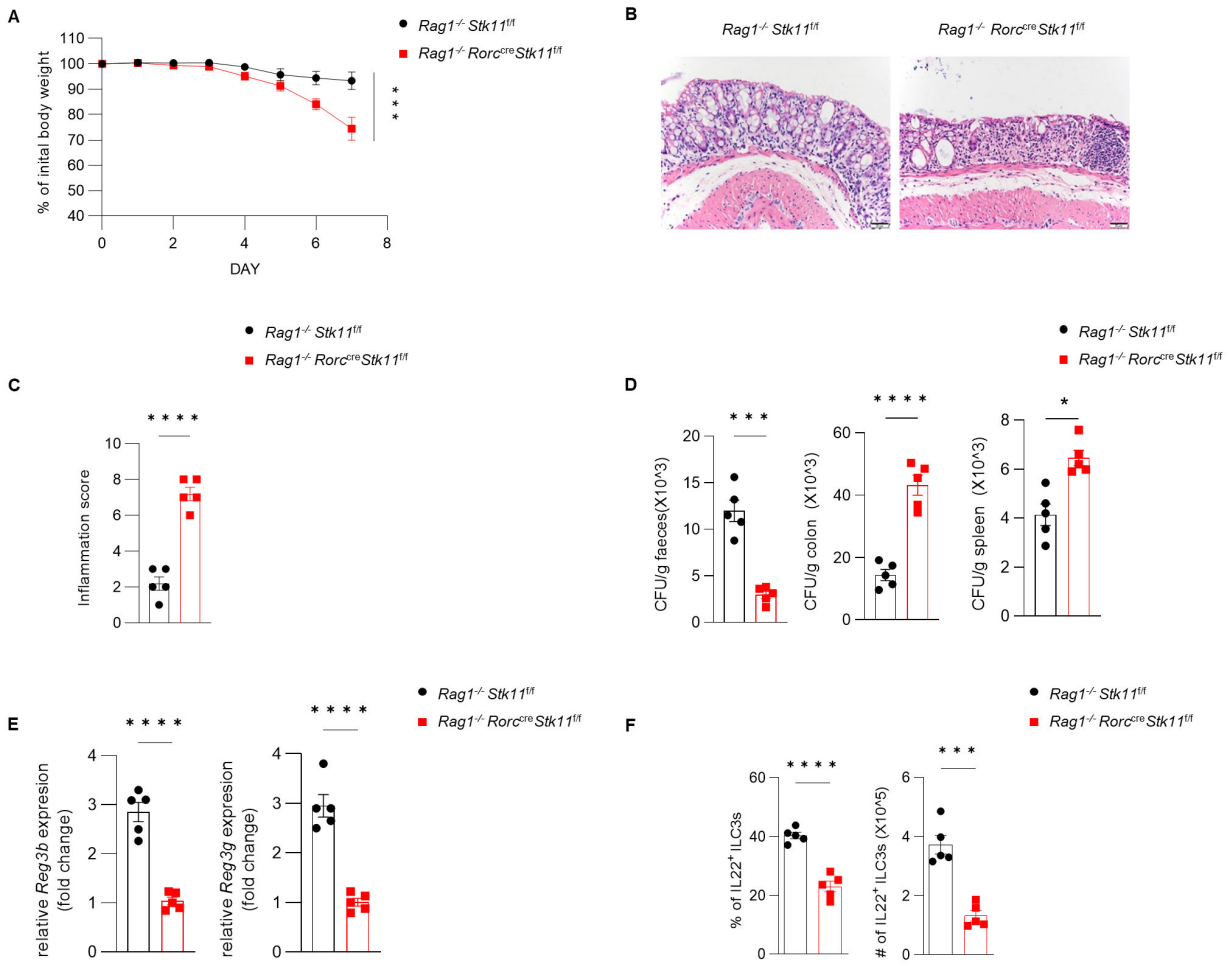
The role of LKB1 in ILC3s in protecting against *C. rodentium* infection is dependent on ILC3s. **(A–D)** 8-week-old *Rag1* ^-/-^ *Stk11^ΔRorc^* mice and their wild-type *Rag1* ^-/-^ *Stk11* ^f/f^ littermates were orally inoculated with *C. rodentium* (2*10^9^ CFU) and analyzed on day 7 after infection. **(A)** Body-weight change (n = 5). **(B)** Histological analysis of representative colons from *Rag1* ^-/-^ *Stk11* ^f/f^ and *Rag1* ^-/-^ *Stk11^ΔRorc^* mice. Scale bars represent 50 mm. **(C)** Histological inflammation scoring of colonic tissues from *Rag1* ^-/-^ *Stk11* ^f/f^ and *Rag1* ^-/-^ *Stk11^ΔRorc^* mice. **(D)** *C. rodentium* titers in feces, colon, and spleen from mice (n = 5). **(E)** Frequency and absolute numbers of IL-22^+^ILC3s in large intestine (LI) from *Stk11* ^f/f^ and *Stk11^ΔRorc^* mice (n = 5). **(F)** The mRNA expression of antimicrobial proteins *Reg3g* and *Reg3b* in the colon of mice (n = 5). Data are representative of three **(A-E)** independent experiments shown as mean ± SEM. Statistical significance was tested by two way ANOVA followed by Tukey’s multiple comparisons test in **(A)**, or two-tailed unpaired Student’s *t*-test in **(C-F)**. *P < 0.05, ***P < 0.001, ****P < 0.0001.

Since ILC3s consist of heterogeneous subpopulations, including Nkp46^+^ ILC3s, Nkp46^-^CCR6^-^ double-negative (DN) ILC3s, and CCR6^+^ lymphoid tissue inducer (LTi)-like ILC3s, we examined whether LKB1 deletion affects the composition of ILC3 subsets. There was no significant difference in large intestine and small intestine lamina propria ILC3 subsets between *Stk11* ^f/f^ and *Stk11^ΔRorc^* mice (**Figure 3A**; **Supplementary Figure S3A**). Given that ILC3s exert effector functions by secreting IL-22 and IL-17, we examined whether LKB1 affects cytokine production in ILC3s. We found that both IL-22 and IL-17 were markedly decreased in intestinal ILC3s from *Stk11^ΔRorc^* mice (**Figures 3B, C**; **Supplementary Figure S3B, C**). These results indicate that LKB1 controls ILC3 effector function. IL-22-expressing ILC3s promote protective immunity against *C. rodentium*, a mouse pathogen that mimics human pathogenic *Escherichia coli* infection (12). Given the reduction of IL-22^+^ ILC3s in *Stk11^ΔRorc^* mice, we next determined the role of LKB1 in gut immunity during *C. rodentium* infection. Compared to *Stk11* ^f/f^ mice, *Stk11^ΔRorc^* mice exhibited increased weight loss and more severe intestinal inflammation (**Figures 4A–C**). Consistently, *Stk11^ΔRorc^* mice also showed lower bacterial loads in the feces and higher bacterial loads in the intestines and spleens (**Figure 4D**). ILC3s maintain colon intestine barrier integrity through IL-22 production, which stimulates the production of antimicrobial peptides by epithelial cells to eliminate pathogenic bacteria. As expected, we observed decreased IL-22 production and reduced expression of antimicrobial peptide-related genes, such as *Reg3b* and *Reg3gr*, in the colon intestine of *Stk11^ΔRorc^* mice (**Figures 4E, F**). These data suggest LKB1 regulates ILC3 function and gut defense against *C. rodentium* infection. To further determine whether LKB1 in ILC3 regulates gut immunity in a cell-intrinsic manner, *Rag1^-^* ^/-^ *Stk11^ΔRorc^* and *Rag1^-^* ^/-^ *Stk11* ^f/f^ control mice were infected with *C. rodentium*. Similar to the findings in *Stk11^ΔRorc^* mice, *Rag1^-^* ^/-^ *Stk11^ΔRorc^* mice exhibited increased intestinal inflammation, more weight loss, and increased bacterial load (**Figures 5A–D**). Consistently, *Rag1^-^* ^/-^ *Stk11^ΔRorc^* mice had reduced IL-22^+^ ILC3s and decreased expression of *Reg3b* and *Reg3g* (**Figures 5E, F**). Collectively, these data suggest that LKB1 regulates ILC3 function in a cell-intrinsic manner.

### 3.5 LKB1 regulates ILC3 cell metabolism

**Figure 6:**
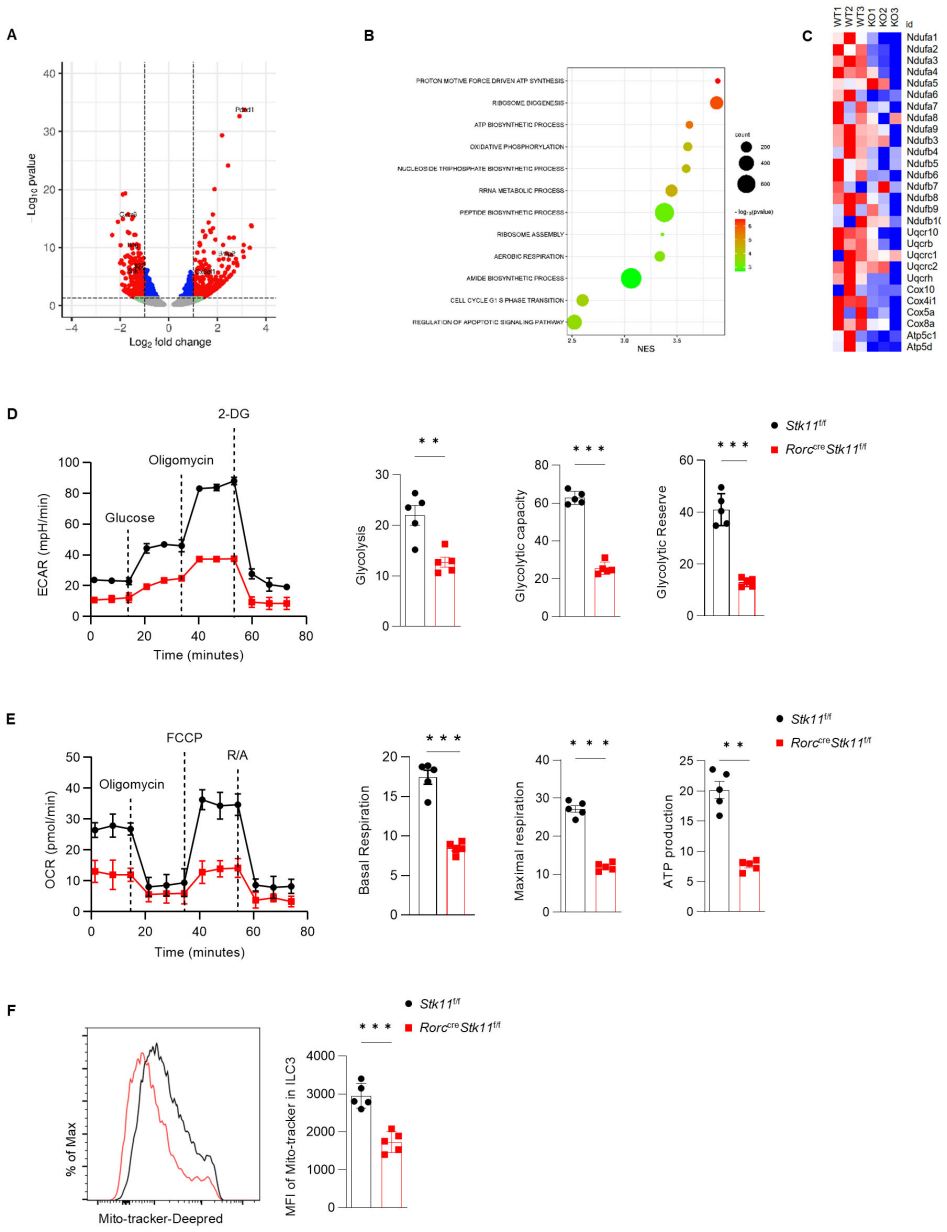
LKB1 supports glycolysis and OXPHOS in ILC3 to maintain cell homeostasis. **(A-C)** RNA-seq analysis of ILC3s in large intestine (LI) from *Stk11* ^f/f^ and *Stk11^ΔRorc^* mice. **(A)** Volcano plot of differentially expressed genes (fold change >= 1.5; P <= 0.05) in ILC3s between *Stk11* ^f/f^ and *Stk11^ΔRorc^* mice. Increased and decreased genes in *Stk11^ΔRorc^* ILC3s were highlighted in red and blue, respectively. **(B)** Gene ontology analysis of RNA-seq data of LI ILC3s from *Stk11* ^f/f^ and *Stk11^ΔRorc^* mice. **(C)** Heatmap of genes associated with mitochondrial respiratory chain complex. **(D, E)** Seahorse analysis of the extracellular acidification rate (ECAR) **(D)** and oxygen consumption rate (OCR) **(E)** in LI ILC3s (n = 5). **(F)** MitoTracker (indicative of mitochondria mass) in LI ILC3s (n = 5). Data are representative of three **(D-F)** independent experiments shown as mean ± SEM. Statistical significance was determined by two-tailed unpaired Student’s t-test in **(D-F)**. **P < 0.01, ***P < 0.001.

To understand the molecular mechanisms by which LKB1 regulates ILC3 survival and function, we performed RNA-sequencing analysis of ILC3s from the large intestine of *Stk11^ΔRorc^* and *Stk11* ^f/f^ mice. LKB1 deletion resulted in 324 upregulated and 250 downregulated significantly differentially expressed genes (DEG) (P value<=0.05, fold change>=1.5) (**Figure 6A**). Gene Ontology (GO) analysis revealed that pathways associated with cell growth, including ribosome biogenesis and assembly, polypeptide synthesis, and cell cycle regulation, were enriched in *Stk11* ^f/f^ mice ILC3s compared to *Stk11^ΔRorc^* mice, which was consistent with the decreased ILC3s number in *Stk11^ΔRorc^* mice (**Figure 6B**). GO also revealed that metabolic pathways, including ATP synthesis and oxidative phosphorylation, were enriched in *Stk11* ^f/f^ mice ILC3s compared to *Stk11^ΔRorc^* mice(**Figure 6B**). By profiling the genes related to mitochondrial metabolism, we found that expression of genes associated with electron transport chain (ETC), such as *Ndufa1* and *Ndufa2*, and genes encoding the enzymes in glycolysis were significantly decreased in *Stk11^ΔRorc^* mice ILC3s (**Figure 6C**; **Supplementary Figure S4A, B**), such as *Atp5c1*. However, genes encoding the metabolic enzymes of the TCA cycle remained unchanged (**Supplementary Figures S4C, D**). These results suggest that LKB1 deficiency might affect both oxidative phosphorylation and glycolysis processes. To functionally link these transcriptional changes to cellular metabolism, we measured the extracellular acidification rate (ECAR) and oxygen consumption rate (OCR) of LKB1-deficient ILC3s. As expected, *Stk11^ΔRorc^* mice ILC3s showed a significant decrease in glycolysis, glycolytic reserve and maximal glycolytic capacity (**Figure 6D**). Additionally, *Stk11^ΔRorc^* mice ILC3s displayed lower basal respiration, maximum respiratory capacity and ATP production. (**Figure 6E**). Furthermore, the reduced oxidative phosphorylation in *Stk11^ΔRorc^* mice ILC3s, correlated to a marked decrease in mitochondrial mass in these cells, while these phenomena were not observed in respective ILC3s from the control mice. To determine whether the reduced oxidative phosphorylation was due to the defect in mitochondria, we examined the mitochondrial mass. *Stk11^ΔRorc^* mice ILC3s showed a marked decrease in mitochondrial mass compared to *Stk11* ^f/f^ mice ILC3s (**Figure 6F**). Together, these findings demonstrate that LKB1 regulates ILC3 cell metabolism intrinsically.

## 4 Discussion

ILC3s play essential roles in maintaining intestinal homeostasis and host defense against enteric infections. However, how ILC3 activation is regulated remains largely unknown. In this study, we demonstrate that LKB1 regulates intestinal ILC3 postnatal development, maintenance, effector function, and cell metabolism. Thus, LKB1 is an important regulator for intestinal immunity.

As a key metabolic regulator, LKB1 has been shown to control cell metabolism and effector function in various immune cell types, including Treg, Th17, macrophages, dendritic cell, and HSC (17–20, 24). Our recent studies show that LKB1 is required to sustain ILC2 mitochondrial fitness by inhibiting PD-1 expression and mitophagy (21). Consistent with the findings in ILC2s, LKB1 regulated ILC3 mitochondria metabolism as well, as indicated by decreased oxidative phosphorylation and less mitochondrial mass. Hence, the decreased IL-22 production in LKB1-deficient ILC3s are presumably due to the impaired energy metabolism. However, the upstream and downstream molecular mechanism through which LKB1 regulates ILC3 metabolism remains unclear, which needs further investigation.

Although LKB1 did not affect ILC3 differentiation during fetal development stage, LKB1 is required for postnatal intestinal ILC3 development. The reduction of intestine ILC3 in LKB1-deficent mice occurred from 2 weeks after birth, and continued at 3-weeks old. Although the time of ILC3 reduction coincided with gut microbiota expansion, our data showed that LKB1 regulation of ILC3 was not dependent on commensal microbiota. These results raised a possibility that other environmental cues might contribute to this process, but this needs to be investigated. To identify the signals which can activate LKB1 in ILC3s during postnatal stage, future work need to be investigated.

In summary, LKB1 plays an important role in linking metabolism and cell fate in ILC3s and ILC3s to ensure their proper development and effector function to mediate intestinal immunity against inflammation and infection. These findings not only advance our knowledge of ILC3 biology but also provide insights into potential therapeutic strategies by targeting LKB1 signaling in intestinal inflammatory diseases.

## Data Availability

The data presented in the study are deposited in the NCBI GEO repository, accession number GSE284792.

## Supplementary material

Supplementary Figure 1 LKB1 is required for intestinal ILC3 homeostasis, related to **Figure 1**. **(A)** Gating strategy for flow sorting large intestine (LI) ILC3s in mice. ILC3s were gated on live and single lymphocytes with Lin^-^ CD45^mid^ CD90^hi^. Lin = CD3e, Gr1, CD11b, CD11c, CD5, CD19, and NK1.1. **(B)** Gating strategy for flow analysis of LI ILC3s in mice. ILC3s were gated on live and single lymphocytes with CD45^+^ Lin^-^ CD127^+^ Rorγt^+^.

Supplementary Figure 2 LKB1 is required for intestinal ILC3 homeostasis, related to **Figure 1**. **(A, B)** Frequency and absolute numbers of ILC3s in small intestine (SI) from *Stk11* ^f/f^ and *Stk11^ΔRorc^* mice (n = 4) **(A)**, or from *Id2* ^cre−ERT2^ and *Id2* ^cre−ERT2^ *Stk11* ^f/f^ mice (n = 5) **(B)**. Cells were gated on live CD45^+^ Lin^-^ CD127^+^ cells **(A, B)**. Data are representative of three **(A, B)** independent experiments shown as mean ± SEM. Statistical significance was tested by two-tailed unpaired Student’s *t*-test in **(A, B)**. *P < 0.05, ***P < 0.001.

Supplementary Figure 3 Ablation of LKB1 in ILC3s results in diminished cytokine production, related to **Figure 3**. **(A)** Frequency and absolute numbers of NKp46^+^ and CCR6^+^ ILC3s in small intestine (SI) (n = 5). **(B, C)** Frequency and absolute numbers of IL-22^+^ILC3s **(B)** and IL-17^+^ILC3s **(C)** in SI (n = 5). Cells were gated on live CD45^+^ Lin^-^ RORγt^+^ lymphocytes **(A-C)**. Data are representative of three **(A-C)** independent experiments shown as mean ± SEM. Statistical significance was tested by two-tailed unpaired Student’s *t*-test in **(A-C)**. **P < 0.01, ***P < 0.001.

Supplementary Figure 4 LKB1 supports glycolysis and OXPHOS in ILC3s to maintain cell homeostasis, related to **Figure 6**. **(A, C)** RNA-seq analysis of ILC3s isolated from the large intestine (LI) from *Stk11* ^f/f^ and *Stk11^ΔRorc^* mice. **(A, C)** Heatmap showing gene expression levels of key enzymes involved in glycolysis (A) and the tricarboxylic acid (TCA) cycle **(C)**. **(B, D)** mRNA expression levels of these key enzymes determined by qRT-PCR. Data are representative of three **(B, D)** independent experiments shown as mean ± SEM. Statistical significance was tested by two-tailed unpaired Student’s *t*-test in **(B, D)**. *P < 0.05, ***P < 0.001.
