# Supplementary figures and images for: LKB1 regulates ILC3 postnatal development and effector function through metabolic programming
Source: Front Immunol. 2025 Jun 5;16:1587256. doi: 10.3389/fimmu.2025.1587256 (PMC12176730; doi:10.3389/fimmu.2025.1587256)

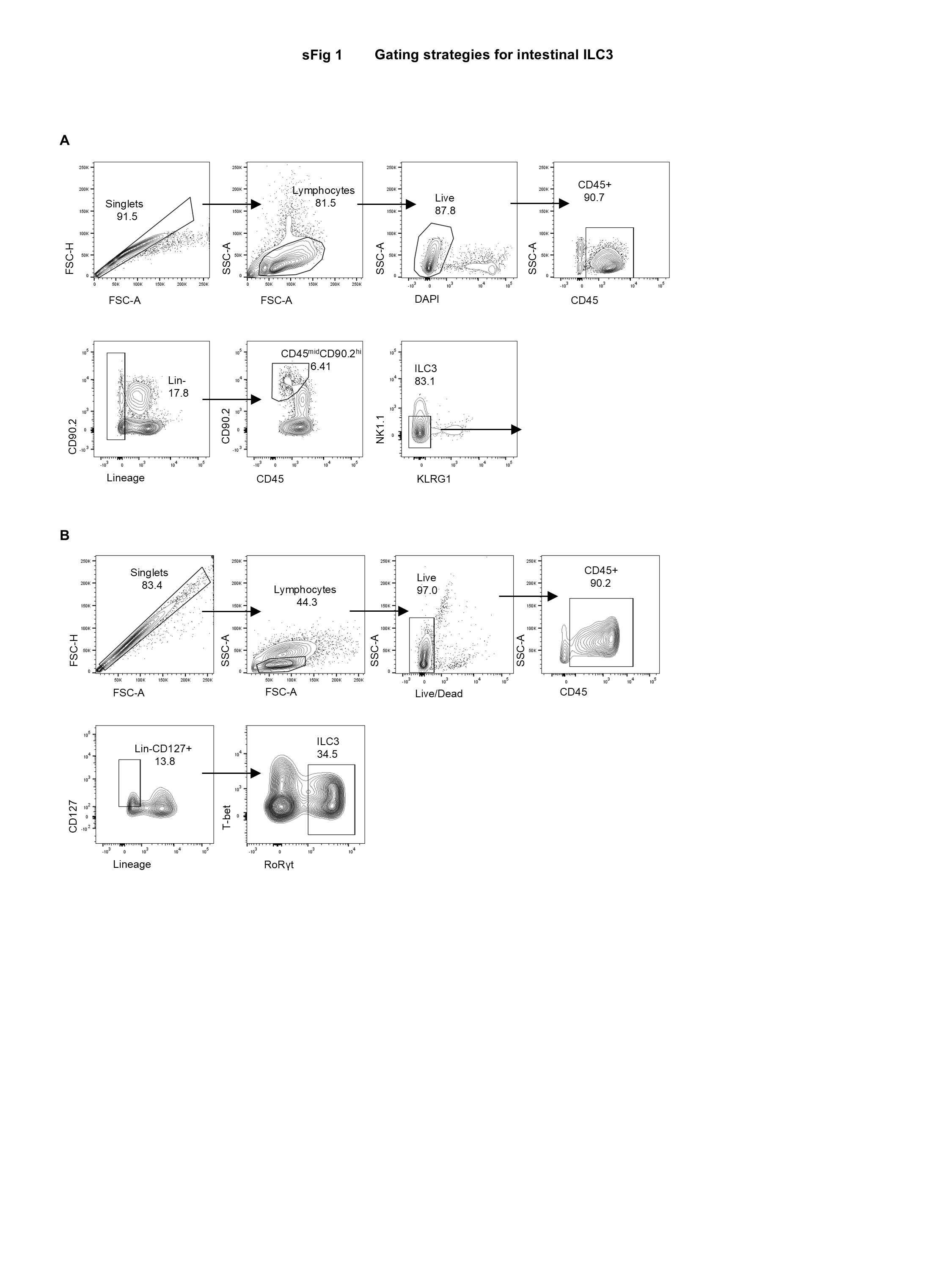

Supplement: Supplementary Figure 1 — LKB1 is required for intestinal ILC3 homeostasis, related to Figure 1 . (A) Gating strategy for flow sorting large intestine (LI) ILC3s in mice. ILC3s were gated on live and single lymphocytes with Lin- CD45mid CD90hi. Lin = CD3e, Gr1, CD11b, CD11c, CD5, CD19, and NK1.1. (B) Gating strategy for flow analysis of LI ILC3s in mice. ILC3s were gated on live and single lymphocytes with CD45+ Lin- CD127+ Rorγt+. [file Image1.tif]

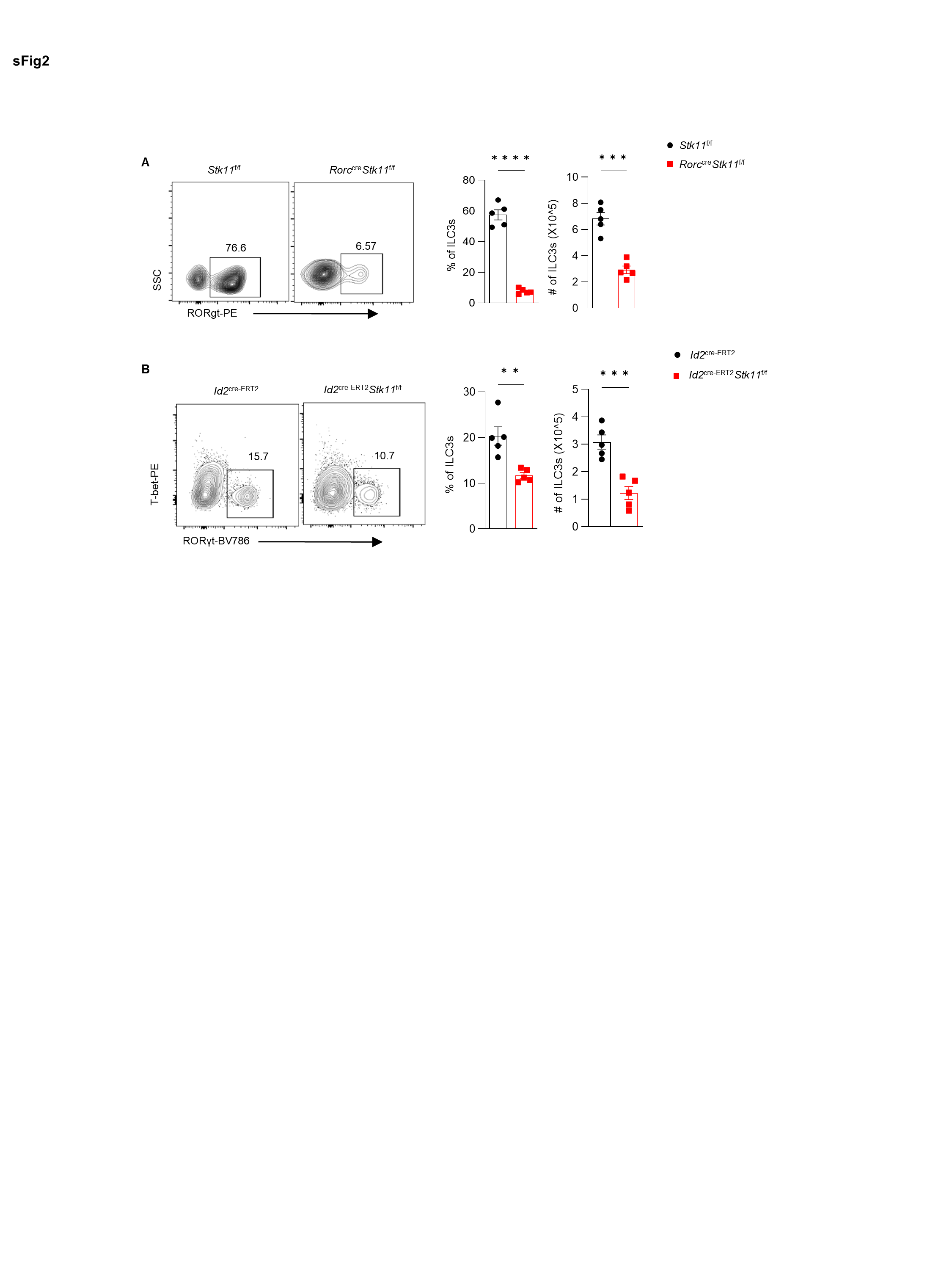

Supplement: Supplementary Figure 2 — LKB1 is required for intestinal ILC3 homeostasis, related to Figure 1 . (A, B) Frequency and absolute numbers of ILC3s in small intestine (SI) from Stk11 f/f and Stk11ΔRorc mice (n = 4) (A), or from Id2 cre−ERT2 and Id2 cre−ERT2 Stk11 f/f mice (n = 5) (B). Cells were gated on live CD45+ Lin- CD127+ cells (A, B). Data are representative of three (A, B) independent experiments shown as mean ± SEM. Statistical significance was tested by two-tailed unpaired Student’s t-test in (A, B). *P < 0.05, ***P < 0.001. [file Image2.tif]

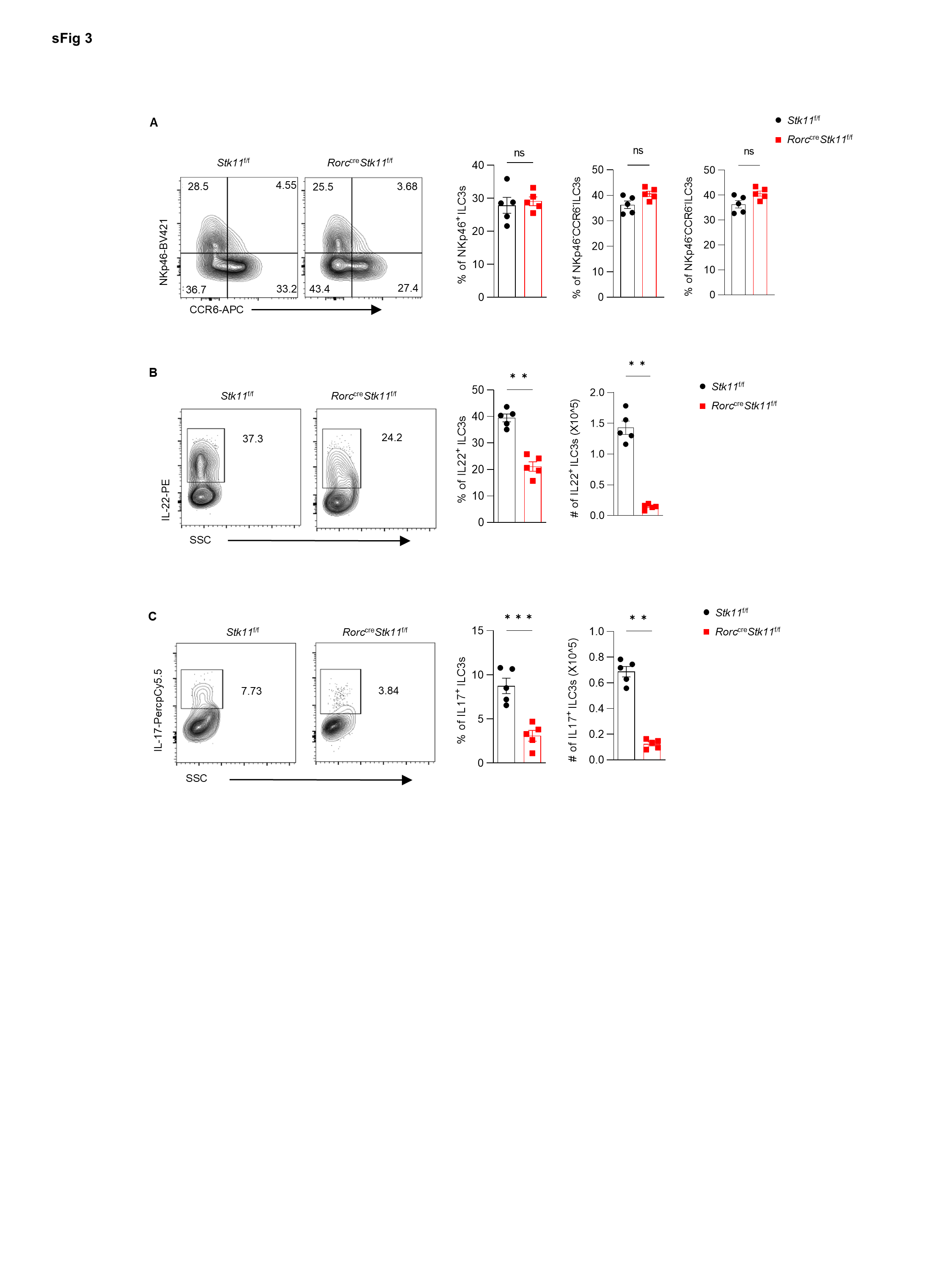

Supplement: Supplementary Figure 3 — Ablation of LKB1 in ILC3s results in diminished cytokine production, related to Figure 3 . (A) Frequency and absolute numbers of NKp46+ and CCR6+ ILC3s in small intestine (SI) (n = 5). (B, C) Frequency and absolute numbers of IL-22+ILC3s (B) and IL-17+ILC3s (C) in SI (n = 5). Cells were gated on live CD45+ Lin- RORγt+ lymphocytes (A-C). Data are representative of three (A-C) independent experiments shown as mean ± SEM. Statistical significance was tested by two-tailed unpaired Student’s t-test in (A-C). **P < 0.01, ***P < 0.001. [file Image3.tif]

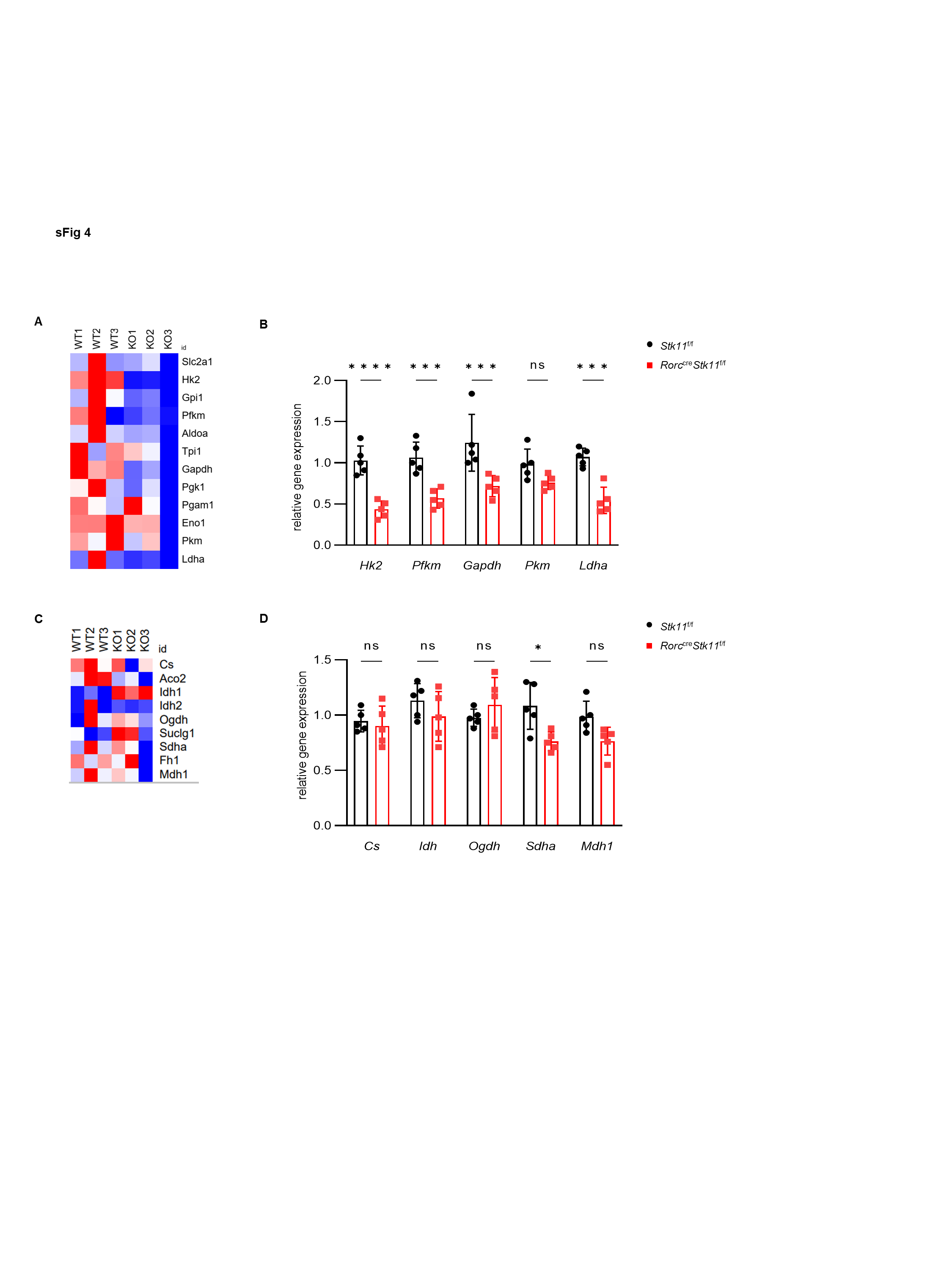

Supplement: Supplementary Figure 4 — LKB1 supports glycolysis and OXPHOS in ILC3s to maintain cell homeostasis, related to Figure 6 . (A, C) RNA-seq analysis of ILC3s isolated from the large intestine (LI) from Stk11 f/f and Stk11ΔRorc mice. (A, C) Heatmap showing gene expression levels of key enzymes involved in glycolysis (A) and the tricarboxylic acid (TCA) cycle (C). (B, D) mRNA expression levels of these key enzymes determined by qRT-PCR. Data are representative of three (B, D) independent experiments shown as mean ± SEM. Statistical significance was tested by two-tailed unpaired Student’s t-test in (B, D). *P < 0.05, ***P < 0.001. [file Image4.tif]
